# Hip Fracture Care during COVID-19: Evolution through the Pandemic

**DOI:** 10.7759/cureus.42696

**Published:** 2023-07-30

**Authors:** Sanjit R Konda, Garrett W Esper, Ariana T Meltzer-Bruhn, Abhishek Ganta, Kenneth A Egol

**Affiliations:** 1 Orthopedic Surgery, Jamaica Hospital Medical Center, New York, USA; 2 Orthopedic Surgery, NYU (New York University) Langone Health, New York, USA; 3 Medical School, Perelman School of Medicine at the University of Pennsylvania, Philadelphia, USA

**Keywords:** orthopedic trauma during covid-19, hospital quality measures, impact on healthcare systems, outcomes, mortality, vaccine, covid-19, hip fracture

## Abstract

Introduction: The purpose of this epidemiologic study was to analyze the care provided by our institution to middle-aged and geriatric hip fracture patients throughout the pandemic to examine for any differences compared to pre-pandemic care and across the pandemic stages.

Methods: Consecutive patients >55 years old treated for hip fractures at our institution between October 2014 and January 2022 were analyzed for demographics, coronavirus disease 2019 (COVID-19) and vaccination status at admission, injury characteristics, hospital quality measures, and outcomes. Patients were divided into three separate cohorts: Pre-COVID-19 (PRECOV), COVID-19 Pre-Vaccine (PREVAX), and COVID-19 Post-Vaccine (POSTVAX). A sub-analysis removed COVID-19-positive patients across the study period. Comparative analyses were conducted.

Results: A total of 2,633 hip fracture patients were included. For the overall cohort, there was no difference in the rate of inpatient deaths between the PRECOV, PREVAX, and POSTVAX cohorts (p=0.278). PRECOV had a significantly lower 30-day mortality rate compared to PREVAX or POSTVAX (p=0.012). Differences in complication rates for surgical site infection, urinary tract infection, and anemia (p<0.01 for all) were seen between cohorts. PRECOV had the longest length of hospital stay (p<0.01). PREVAX patients required more ICU level of care (p<0.01). When removing COVID-19-positive patients, all three cohorts had similar inpatient (p=0.872) and 30-day mortality rates (p=0.130).

Conclusion: The care of patients treated for hip fractures did not change throughout the pandemic at our institution. The elevated mortality rate due to the effects of COVID-19 seen in the pre-vaccine cohort decreased over time as the understanding of COVID-19 improved and the vaccine was introduced. We recommend continuation of the same hip fracture care protocols as used pre-pandemic.

## Introduction

The coronavirus disease 2019 (COVID-19) pandemic has greatly affected the world since it first began at the end of 2019. From the early days of late winter/early spring of 2020 to May 10, 2023, there have been 765,903,278 confirmed cases of COVID-19 globally with 6,927,378 deaths [1]. During the same period, the caseload and death toll in the United States stand at 106,831,286 and 1,163,294, respectively [2]. Over time, the virus has evolved to include new variants such as Delta and Omicron, lending to an evolving challenge with surges in case and death rates occurring periodically since the early months.

The onset of the COVID-19 pandemic had a large impact on healthcare systems and caused hospitals to be stretched for resources. High volumes of infected individuals caused many hospitals to restructure their departments and staffing requirements to increase the availability of beds, staff, and treatments available for COVID-19-infected patients [3-5]. These changes led to significant disruptions in various medical specialties including those at the forefront of the fight against COVID-19 and those not. Orthopedic surgeons were not usually at the front lines of the fight against COVID-19; however, the effects of COVID-19 have had an impact on all aspects of trauma care and orthopedic care [6]. The pandemic has impacted and continues to impact emergency and elective surgeries, trauma volumes, in-person vs. telemedicine visits, outpatient clinics, staffing management, rehabilitation, resident training, and use of personal protective equipment (PPE) among many other areas [5,6]. As the pandemic continued, the medical community evolved to combat COVID-19 more effectively. In orthopedics, various management guidelines were created for surgery scheduling, triage and management of trauma, PPE use, discharge locations, healthcare workers, and patients across the board [5-7]. Research is ongoing into the effects these had on patient care.

The presence of the COVID-19 pandemic coupled with the well-documented risk for morbidity and mortality associated with hip fractures highlights a significant challenge facing providers today [8-10]. Multiple studies from both the initial wave in the spring of 2020 and in the years following have demonstrated that COVID-19 infection is associated with worse functional outcomes and a much higher risk of mortality out through the one-year mark in the middle-aged and geriatric populations [11-13]. Therefore, it was paramount that these patients received timely treatment whether it be through surgical fixation or medical management in order to maintain our high standards of care even during the pandemic to minimize the risk of morbidity and mortality in these vulnerable populations.

The development of the vaccine by the end of 2020 marked a significant timepoint in the pandemic’s evolution, allowing providers to combat the acute effects of COVID-19 infection more effectively. Multiple studies have demonstrated the vaccine’s efficacy in mitigating the occurrence of admission, inpatient complications, and deaths secondary to COVID-19 infection [14,15].

The purpose of this epidemiologic study was to analyze the care provided by one multi-center institution for patients treated for hip fractures during the pandemic with their subsequent outcomes, examining potential differences with pre-pandemic care and across the different stages of the pandemic.

## Materials and methods

New York University (NYU) Langone Health Office of Science and Research Institutional Review Board (approval number: i20-01766) approved the use of a geriatric trauma database to review all patients, aged 55 or older, who sustained a hip fracture via a low-energy mechanism of injury between October 2014 and January 2022 (low-energy was defined as a fall from standing or from a height <2 stairs). The study was conducted at NYU Langone Health and Jamaica Hospital Medical Center, New York, United States. All patients were treated at one academic medical center that included three Level 1 trauma centers, one university-based tertiary care referral hospital, and one orthopaedic specialty hospital.

All centers within our institution followed a similar four-day hip fracture treatment protocol [16-18]. For this study, patient injuries were classified based on Arbeitsgemeinschaft für Osteosynthesefragen/Orthopaedic Trauma Association (AO/OTA) classifications and included intertrochanteric (AO/OTA 31A), femoral neck (AO/OTA 31B), and subtrochanteric (AO/OTA 32(A-C)) hip fractures [19]. Exclusion criteria were patients younger than 55 and/or those who did not sustain their injury through a low-energy mechanism.

The hip fracture protocol utilized within this study included medicine co-management of all patients and concrete, well-delineated tasks for all members of the care team including nurses, doctors, social workers, and patient care managers, as well as both physical and occupational therapists. The goals of this protocol include immediate medical preparation for surgery followed by rapid surgical intervention with early identification of discharge barriers and coordination of physical therapy and occupational therapy for rehabilitation purposes. Likewise, at their time of arrival at the Emergency Department (ED), patients were rapidly screened by the initial treatment team and evaluated for medical clearance. If medically appropriate, patients were then transferred to a tertiary care orthopedic hospital with hospitalist co-management.

Demographics were collected for each patient that included age, sex, body mass index (BMI), sex, hip fracture classification per AO/OTA guidelines, baseline ambulatory/functional status, and comorbidities as represented by the Charlson Comorbidity Index (CCI) [16]. Injury presentation variables collected were Glasgow Coma Scale (GCS) and Abbreviated Injury Severity scores (AIS) for head/neck (AIS H/N) and chest (AIS C). Patients were identified as COVID-19 positive if they had a positive COVID-19 RT-PCR test at the time of admission to the hospital for their hip fracture. All COVID-19 patients included in this analysis were symptomatic. Patients who had received their first dose (Janssen/Pfizer/Moderna) of the COVID-19 vaccine prior to admission were classified as COVID-19 vaccinated.

Hospital quality measures collected for each patient included length of stay (LOS) and need for intensive care unit (ICU) level of care. Complications recorded during each patient’s index hospitalization included sepsis/septic shock, pneumonia, deep vein thrombus/pulmonary embolism (DVT/PE), myocardial infarction (MI), acute renal failure/acute kidney injury (ARF/AKI), stroke, surgical site infection (SSI), decubitus ulcer, urinary tract infection (UTI), acute respiratory failure (ARF), anemia, and cardiac arrest. Mortality measures collected included inpatient mortality and mortality within 30 days of discharge [15].

Demographic data were characterized through descriptive summary statistics. Continuous variables were compared with the Mann-Whitney U test. Categorical variables were compared with chi-square test. Comparison of both categorical and continuous variables was done using independent sample t-tests and ANOVA. Patients were divided into three separate cohorts: Pre-COVID-19 (PRECOV), COVID-19 Pre-Vaccine (PREVAX), and COVID-19 Post-Vaccine (POSTVAX). The POSTVAX time period was defined as after December 1, 2020, to capture the period once the first vaccine was publicly available for the United States. Statistics were calculated with IBM SPSS Statistics for Windows, Version 25.0 (Released 2017; IBM Corp., Armonk, New York, United States). Significance was defined with an alpha of 0.05.

## Results

**Figure 1:**
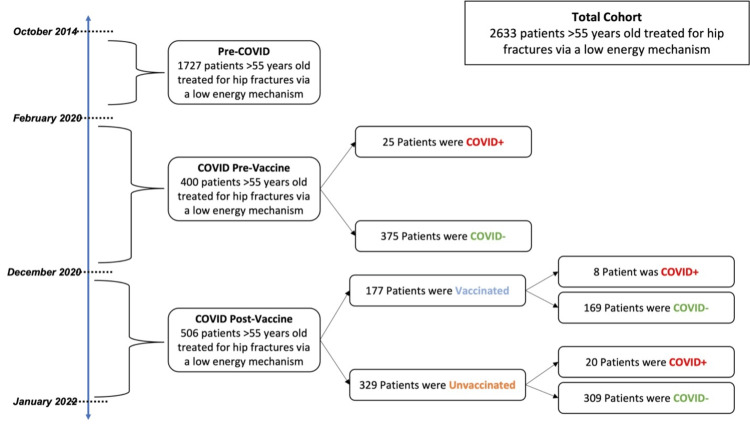
Demographic breakdown of patient cohorts COVID=COVID-19 (coronavirus disease 2019); COVID+= coronavirus disease 2019 positive

Between October 2014 and January 2022, 2633 patients aged 55 years and older who sustained a hip fracture via a low-energy mechanism were seen in one healthcare system and enrolled in an IRB-approved geriatric trauma database. The PRECOV cohort included 1727 patients seen between October 2014 and February 2020. The PREVAX cohort included 400 hip fracture patients who presented after the onset of the pandemic (February 1, 2020) but before the first administration of the COVID-19 vaccine (December 14, 2020). The POSTVAX cohort included 506 hip fracture patients that presented after the first vaccine administration (Figure 1).

Population characteristics for the overall patient cohort demonstrate 69% female sex, mean age of 80.63 years ± 10.34, mean BMI of 24.19 ± 4.94, mean GCS of 14.87 ± 0.62, mean CCI of 1.49 ± 1.74, mean AIS H/N of 0.03 ± 0.28, and mean AIS C of 0.02 ± 0.19. Within the pandemic cohorts (PREVAX and POSTVAX), 53 patients (5.8%) were COVID-19-positive, and 177 (19.5%) patients were vaccinated with at least one dose at the time of admission. Patients’ ambulatory status at admission included: 1777 (67%) were community ambulators, 751 (29%) were household ambulators, and 105 (4%) were non-ambulatory. The most common AO/OTA fracture classifications included: 1397 (53%) 31A, 1138 (43%) 31B. The most common interventions included: short intramedullary nail (n=1051; 40%) and hemiarthroplasty (n=595; 23%).

Differences between the PRECOV, PREVAX, and POSTVAX cohorts included a larger proportion of community ambulators in the PRECOV cohort (70.53% vs 58.00% vs 64.62%, respectively; p<0.01) and a larger proportion of household ambulators in the PREVAX cohort (25.77% vs 37.00% vs 31.23%, respectively; p<0.01). Significant differences were also seen in regard to the fracture classifications and treatments chosen throughout the pandemic (Table 1).

**Table 1:** Demographics for the three cohorts GCS=Glascow Coma Scale; CCI=Charlson Comorbidity Index; AIS=Abbreviated Injury Score; CRPP=Closed Reduction Percutaneous Pinning; IMN=Intramedullary Nail; COVID-19=coronavirus disease 2019 *Statistically significant

Variables	Pre-COVID-19	COVID-19 Pre-Vaccine	COVID-19 Post-Vaccine	Total	P-Value
Demographics	N (%)	N (%)	N (%)	N (%)	
Total patients	1727	400	506	2633	
Age (years; mean ± std)	80.76 ± 10.19	80.80 ± 10.31	80.03 ± 10.85	80.63 ± 10.34	0.354
BMI (kg/m^2^, mean ± std)	24.18 ± 6.75	24.31 ± 5.76	24.11 ± 5.45	24.19 ± 4.94	0.829
Gender, n (%)					0.016*
Male	502 (29.07%)	144 (36.00%)	165 (32.61%)	811 (30.80%)	
Female	1225 (70.93%)	256 (64.00%)	341 (67.39%)	1822 (69.20%)	
Ambulatory Status, n (%)					<0.01*
Community ambulator	1218 (70.53%)	232 (58.00%)	327 (64.62%)	1777 (67.49%)	<0.01*
Household ambulator	445 (25.77%)	148 (37.00%)	158 (31.23%)	751 (28.52%)	<0.01*
Non-ambulatory	64 (3.71%)	20 (5.00%)	21 (4.15%)	105 (3.99%)	0.271
COVID-19 +, n (%)	0 (0.00%)	25 (6.25%)	28 (5.53%)	53 (2.01%)	-
COVID-19 vaccinated, n (%)	0 (0.00%)	0 (0.00%)	177 (34.98%)	177 (6.72%)	-
Medical Characteristics (mean ± std)					
GCS	14.88 ± 0.55	14.85 ± 0.80	14.83 ± 0.67	14.87 ± 0.62	0.205
CCI	1.50 ± 1.75	1.46 ± 1.65	1.51 ± 1.74	1.49 ± 1.74	0.895
AIS head/neck	0.03 ± 0.25	0.04 ± 0.32	0.05 ± 0.34	0.03 ± 0.28	0.565
AIS chest	0.02 ± 0.17	0.02 ± 0.19	0.02 ± 0.23	0.02 ± 0.19	0.998
Fracture Classification, N (%)					<0.01*
31A	914 (52.92%)	207 (51.75%)	276 (54.55%)	1397 (53.06%)	0.484
31B	727 (42.10%)	184 (46.00%)	227 (44.86%)	1138 (43.22%)	0.110
32A	53 (3.07%)	8 (2.00%)	3 (0.59%)	64 (2.43%)	<0.01*
32B	2 (0.12%)	1 (0.25%)	0 (0.00%)	3 (0.11%)	0.368
32C	31 (1.80%)	0 (0.00%)	0 (0.00%)	31 (1.18%)	<0.01*
Treatment, N (%)					<0.01*
CRPP	141 (8.16%)	37 (9.25%)	50 (9.88%)	228 (8.66%)	0.230
Hemiarthroplasty	387 (22.41%)	101 (25.25%)	107 (21.15%)	595 (22.60%)	0.162
Total hip arthroplasty	113 (6.54%)	18 (4.50%)	36 (7.11%)	167 (6.34%)	0.110
Long IMN	236 (13.67%)	68 (17.00%)	44 (8.70%)	348 (13.22%)	<0.01*
Short IMN	711 (41.17%)	136 (34.00%)	204 (40.32%)	1051 (39.92%)	0.01*
Sliding hip screw	86 (4.98%)	11 (2.75%)	9 (1.78%)	106 (4.03%)	<0.01*
Nonoperative	48 (2.78%)	26 (6.50%)	29 (5.73%)	103 (3.91%)	<0.01*
Transferred	8 (0.46%)	4 (1.00%)	2 (0.40%)	14 (0.53%)	0.643

Comparative epidemiologic study of the three cohorts

There was no difference in the rate of inpatient deaths between the three cohorts (1.80% vs. 3.00% vs. 2.57%; p=0.278). The PRECOV cohort had a significantly lower 30-day mortality rate compared to the PREVAX or POSTVAX cohorts (PRECOV 4.23% vs. PREVAX 7.75% vs. POSTVAX 5.14%; p=0.012). Patients who were COVID-19-positive in the PREVAX cohort experienced a higher rate of inpatient (24.00% vs 14.29%, p=0.087) and 30-day mortality (28.00% vs 21.43%, p=0.378) as compared to COVID-19-positive patients in the POSTVAX cohort; however, this difference was not significant.

Among inpatient complications, there were significant differences in rates of surgical site infection (0.06% vs 0.25% vs 1.19%; p<0.01), UTI (8.98% vs. 5.75% vs. 5.14%; p<0.01), and anemia (32.66% vs. 24.75% vs. 25.69%; p<0.01) between the three cohorts. The PRECOV cohort had the longest length of hospital stay (PRECOV 6.72 days ± 4.25 vs. PREVAX 6.09 days ± 4.70 days vs. POSTVAX 6.00 days ± 5.75; p<0.01). The PREVAX cohort had a significantly higher percentage of patients need ICU-level care (PRECOV 18.93% vs. PREVAX 24.00% vs. POSTVAX 11.86%; p<0.01) (Table 2).

**Table 2:** Comparison of the outcomes of the three cohorts DVT/PE=Deep Vein Thrombosis/Pulmonary Embolism; MI=Myocardial Infarction; AKI=Acute Kidney Injury; SSI=Surgical Site Infection; UTI=Urinary Tract Infection; ARF=Acute Respiratory Failure; LOS=Length of Stay *Statistically significant

Outcomes	Pre-COVID (n=1727)	COVID-19 Pre-Vaccine (n=400)	COVID-19 Post-Vaccine (n=506)	P-Value
Complications, n (%)				
Sepsis/Septic Shock	39 (2.26%)	9 (2.25%)	16 (3.16%)	0.979
Pneumonia	78 (4.52%)	25 (6.25%)	22 (4.35%)	0.304
DVT/PE	35 (2.03%)	7 (1.75%)	8 (1.58%)	0.798
MI	22 (1.27%)	5 (1.25%)	3 (0.59%)	0.445
AKI	138 (7.99%)	31 (7.75%)	47 (9.29%)	0.563
Stroke	7 (0.41%)	4 (1.00%)	2 (0.40%)	0.294
SSI	1 (0.06%)	1 (0.25%)	6 (1.19%)	<0.01*
Decubitus Ulcer	25 (1.45%)	4 (1.00%)	4 (0.79%)	0.455
UTI	155 (8.98%)	23 (5.75%)	26 (5.14%)	<0.01*
ARF	82 (4.75%)	23 (5.75%)	22 (4.35%)	0.613
Anemia	564 (32.66%)	99 (24.75%)	130 (25.69%)	<0.01*
Cardiac Arrest	19 (1.10%)	8 (2.00%)	6 (1.19%)	0.343
Hospital Quality Measures				
LOS (days, mean ± std)	6.72 ± 4.25	6.09 ± 4.70	6.00 ± 5.75	<0.01*
Need for ICU, n (%)	327 (18.93%)	96 (24.00%)	60 (11.86%)	<0.01*
Discharged Home, n (%)	363 (21.02%)	115 (28.75%)	162 (32.02%)	<0.01*
Mortality, n (%)				
Inpatient	31 (1.80%)	12 (3.00%)	13 (2.57%)	0.278
Within 30 days	73 (4.23%)	31 (7.75%)	26 (5.14%)	0.012*
Total mortality within COVID-19 + populations	-	25	28	
Inpatient, n (%)	-	6 (24.00%)	4 (14.29%)	0.087
Within 30 days, n (%)	-	7 (28.00%)	6 (21.43%)	0.378

**Figure 2:**
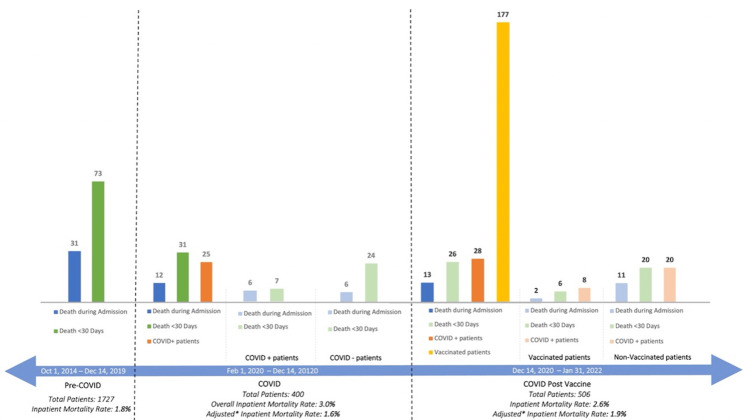
Comparison of patient mortality rates throughout the pandemic COVID=COVID-19 (coronavirus disease 2019)

A sub-analysis was conducted that removed COVID-19-positive patients to examine baseline hip fracture care amongst the three cohorts. There was no difference in inpatient mortality rates (PRECOV 1.80% vs. PREVAX 1.60% vs. POSTVAX 1.88%; p=0.872) and 30-day mortality rates (PRECOV 4.23% vs PREVAX 6.40% vs POSTVAX 4.18%; p=0.130) (Figure 2).

Among inpatient complications, there were significant differences in rates of surgical site infection (PRECOV 0.06% vs PREVAX 0.00% vs POSTVAX 1.26%; p<0.01), UTI (PRECOV 8.98% vs. PREVAX 5.60% vs. POSTVAX 5.23%; p<0.01), and anemia (PRECOV 32.66% vs. PREVAX 23.47% vs. POSTVAX 24.90%; p<0.01) between the three cohorts. PRECOV cohort had the longest LOS (PRECOV 6.72 days ± 4.25 vs. PREVAX 5.84 days ± 3.78 days vs. POSTVAX 5.69 days ± 5.31; p<0.01). The PREVAX cohort had a significantly higher percentage of patients that need ICU-level care (PRECOV 18.93% vs. PREVAX 23.73% vs. POSTVAX 11.72%; p<0.01) (Table 3).

**Table 3:** Comparison of outcomes the cohorts controlling for patients that tested positive for COVID-19 on admission DVT/PE=Deep Vein Thrombosis/Pulmonary Embolism; MI=Myocardial Infarction; AKI=Acute Kidney Injury; SSI=Surgical Site Infection; UTI=Urinary Tract Infection; ARF=Acute Respiratory Failure; LOS=Length of Stay; COVID-19=Coronavirus Disease 2019 *Statistically significant

Outcomes without COVID	Pre-COVID-19	COVID-19 Pre-Vaccine	COVID-19 Post-Vaccine	P-Value
	n (%)	n (%)	n (%)	
N	1727	375	478	
Complications				
Sepsis/Septic Shock	39 (2.26%)	6 (1.60%)	13 (2.72%)	0.676
Pneumonia	78 (4.52%)	10 (2.67%)	16 (3.35%)	0.187
DVT/PE	35 (2.03%)	5 (1.33%)	6 (1.26%)	0.418
MI	22 (1.27%)	3 (0.80%)	3 (0.63%)	0.417
AKI	138 (7.99%)	28 (7.47%)	42 (8.79%)	0.733
Stroke	7 (0.41%)	4 (1.07%)	2 (0.42%)	0.251
SSI	1 (0.06%)	0 (0.00%)	6 (1.26%)	<0.01*
Decubitus Ulcer	25 (1.45%)	4 (1.07%)	4 (0.84%)	0.540
UTI	155 (8.98%)	21 (5.60%)	25 (5.23%)	<0.01*
ARF	82 (4.75%)	16 (4.27%)	15 (3.14%)	0.325
Anemia	564 (32.66%)	88 (23.47%)	119 (24.90%)	<0.01*
Cardiac Arrest	19 (1.10%)	5 (1.33%)	6 (1.26%)	0.906
Hospital Quality Measures				
LOS (d, mean ± std)	6.72 ± 4.25	5.84 ± 3.78	5.69 ± 5.31	<0.01*
Need for ICU	327 (18.93%)	89 (23.73%)	56 (11.72%)	<0.01*
Discharged Home	363 (21.02%)	112 (29.87%)	152 (31.80%)	<0.01*
Mortality				
Inpatient	31 (1.80%)	6 (1.60%)	9 (1.88%)	0.872
Within 30 days	73 (4.23%)	24 (6.40%)	20 (4.18%)	0.130

## Discussion

In this study, we analyzed one health system's experience with patients treated for hip fractures during the pandemic. Our study found similar rates of inpatient mortality across the entire period of the pandemic, especially when controlling for COVID-19-positive patients. Additionally, this study demonstrates the higher rates of 30-day mortality and the need for ICU admission seen during the early stages of the pandemic abated as the pandemic continued to progress.

The onset of the COVID-19 pandemic rapidly stressed healthcare systems and medical specialties across the globe. While orthopedic surgeons were not usually at the forefront, the effects of COVID-19 have had an impact on all aspects of orthopedic care [6]. Patients who sustained hip fractures within the first months of the pandemic experienced drastically increased rates of inpatient mortality, 30-day, and one-year mortality, longer LOS with higher rates of inpatient complications, and worse functional outcomes [11-13,20-26]. Amidst the already high rates of mortality and morbidity found in hip fracture patients, having a thorough understanding of the impact of COVID-19 on this population is a public health concern.

The overall inpatient death rate rose during the beginning of the pandemic as medical providers were dealing with the unknown (1.8% - >3.0%). However, since the initial surge of cases in the spring of 2020, the medical community’s understanding and management of COVID-19 have improved to combat the virus and subsequent variants [27]. Widespread administration of the COVID-19 vaccine has similarly worked to blunt the impact of the virus on patients and healthcare providers alike [14,28]. Comparing the inpatient death rate of the PREVAX cohort to the POSTVAX cohort, it is clear that care has improved (3.0% -> 2.6%). In the setting of orthopedic trauma, specifically our management of hip fracture patients, this improvement can also be reflected in the lower inpatient death rate seen amongst COVID-19-positive hip fracture patients (24% -> 14%).

The 30-day mortality rate was found to be significantly elevated in the PREVAX cohort. This may be explained by the shorter LOS seen as patients may have been discharged at an earlier stage in their recovery. Patients may have missed inpatient and outpatient physical and occupational therapy sessions that would have been instrumental to their recovery. With the travel restrictions in place as well as what we have seen anecdotally in the early stages of the pandemic, patients were less willing to travel to appointments for fear of infection. Additionally, patients in the PREVAX cohort experienced a change in discharge location similar to those reported in the literature as many facilities restricted which patients they admitted [29]. While potentially beneficial for some as they returned home sooner, many patients may not have been able to receive structured step-wise progressions of rehabilitation. Occupational and physical therapy has been shown to improve overall function and health after hip fractures [30-32]. Without that care and with fewer hours of inpatient supervision, older patients’ outcomes were worse. This corresponds with current literature that shows that patients who did not regain their pre-fracture basic mobility experienced an increased 30-day mortality rate [33]. Furthermore, frailty and advanced age were all associated with an increased risk of postoperative mortality for hip fracture patients [34,35]. Further research is needed to assess the impact COVID-19 had on discharge locations for patients treated for hip fractures, including long-term outcomes.

In order to compare immediate hip fracture care among the three cohorts accurately, we reexamined inpatient mortality without the influence of COVID-19. We found that there was no difference in inpatient mortality between the three cohorts when COVID-19-positive patients were removed. This highlights our institution's continued ability to provide effective hip fracture care to patients, despite the ongoing backdrop of the COVID-19 pandemic. In addition, there was no significant difference in 30-day mortality for any of the three cohorts, demonstrating that even during the COVID-19 pandemic, our patients did not experience a significantly higher mortality rate as compared to pre-pandemic. It is reassuring to see that the high level of care provided for hip fracture patients did not change, even with the disruptions caused by COVID-19.

Patients who presented in the PREVAX cohort were more likely to require an elevated level of care. Our institution experienced an elevated rate (24%) of patients requiring ICU admission during the COVID-19 pandemic prior to the vaccine. This aligns with literature from the early days of the pandemic, demonstrating similar rates of ICU admission for ventilatory support and around-the-clock care in the backdrop of high rates of COVID-19 positivity [36]. While those studies were not based strictly on hip fracture patients, our institutional hip fracture ICU admission rate aligns with those reported in the literature [37]. The increased need for ICU level of care seen in the PREVAX cohort decreases below PRECOV cohort levels in the POSTVAX cohort, demonstrating a decrease in the need for ICU level of care as the pandemic progressed. This initial increase seen in the PREVAX cohort, many of whom presented during the first wave of COVID-19 when hospitals were overwhelmed, may have been due to worse overall inpatient status as the healthcare system struggled to accommodate the sudden influx of very sick COVID-19-positive patients. Additionally, this may have been due to surgeons and care teams having a lower threshold for ICU admission during the early stages of the pandemic given the fear of worsening health status secondary to COVID-19 infection.

This study has several limitations. The data we examined only encompassed one health system’s experience. While our health system is located in a diverse metropolitan area, our patient population may be different from those in other urban or rural areas. Especially during the pandemic, major metropolitan areas were often hotspots for COVID-19, meaning our experience may not be reflective of all places. Additionally, this is a retrospective study. As the pandemic continues to fluctuate and change, our findings may not fully represent the impact of new changes in COVID-19 variants.

## Conclusions

The care of patients treated for hip fractures did not change throughout the pandemic at our institution. The elevated mortality rate due to the effects of COVID-19 seen in the pre-vaccine cohort decreased over time as the understanding of COVID-19 improved and the vaccine was introduced. We recommend the continuation of the same hip fracture care protocols as used in the pre-pandemic period. Additionally, we urge providers in all specialties to recommend vaccination as all geriatric patients at risk for fracture would benefit from COVID-19 vaccination. While many studies have discussed the impact of the COVID-19 pandemic on fracture care, these have all focused on the early months of the pandemic. Therefore, we sought to expand the discussion regarding hip fracture care in a longitudinal scope closer to the present day.
